# Role of the initial degree of anaemia and treatment model in the prognosis of gastric cancer patients treated by chemotherapy: a retrospective analysis

**DOI:** 10.1186/s12885-020-06881-7

**Published:** 2020-05-13

**Authors:** Wen-Huan Li, Ji-Yu Zhang, Wen-Hui Liu, Xian-Xian Chen

**Affiliations:** 1 Department of Oncology, Shandong Provincial Hospital Affiliated to Shandong University, 324 Jingwu RD, Jinan, 250021 Shandong People’s Republic of China; 2 Shandong Center for Diseases Control and Prevention, 16992 Jingshi RD, Jinan, 250014 Shandong People’s Republic of China; 3 School of Public Health, Shandong University, Jinan, 250012 Shandong People’s Republic of China

**Keywords:** Anaemia, Gastric cancer, Chemotherapy, Prognosis, Decrease in haemoglobin

## Abstract

**Background:**

Anaemia is highly prevalent in gastric cancer (GC) patients. The role of initial haemoglobin levels in predicting the prognosis of GC patients treated by chemotherapy has not been well determined. Our present study aims to evaluate the relationship between the degree of anaemia and the overall survival (OS) and progression-free survival (PFS) of patients with GC.

**Methods:**

Our retrospective study enrolled 598 patients who were treated with chemotherapy when the recurrent or metastatic GCs were unsuitable for surgical resection. Univariate and multivariate analyses were performed to identify risk factors that had the potential to affect patient prognosis. Additionally, the relationship between clinicopathological characteristics, including treatment method, and degree of cancer-related reduction in haemoglobin was further analysed.

**Results:**

Our results revealed that patients with HB_ini_ level ≤ 80 g/L had a trend toward a shortened median OS and PFS (*p* = 0.009 and *p* = 0.049, respectively). Interestingly, we also found that HB_dec_ ≥ 30 g/L was associated with a significantly shortened median OS and PFS (*p* = 0.039 and *p* = 0.001, respectively). Multivariate analysis showed that HB_ini_ levels ≤80 g/L could be used as an independent prognostic factor for recurrent and metastatic GC. More importantly, HB_dec_ ≥ 30 g/L and treatment response were also significantly associated with OS and PFS. Furthermore, the degree of haemoglobin decrease was associated with chemotherapy including platinum and the number of chemotherapy cycles.

**Conclusion:**

Our study concludes that the initial degree of anaemia and a decrease in haemoglobin of ≥30 g/L can serve as biomarkers to predict prognosis in recurrent or metastatic GC patients, while chemotherapy treatment rather than red blood cell (RBC) transfusion can improve their prognosis. Additionally, platinum should not be recommended for treating severely anaemic GC patients.

## Background

Gastric cancer (GC) is the fifth most common malignant tumour and the third leading cause of death worldwide [1]. Recurrence and metastasis are the most important characteristics of cancers including GC [2, 3]. The incidence of anaemia in advanced gastric cancer patients is high, with a large variability ranging from 10 to 30% [4, 5]. Anaemia can weaken the fragile patient and has been reported to be associated with a poor clinical outcome. However, the role of the degree of anaemia and treatment model in recurrent or metastatic GC patient prognosis is unclear. Therefore, managing and improving the condition of GC-related anaemia through medical approaches are urgently needed to improve the prognosis of patients with recurrent or metastatic GC.

Cancer-related anaemia (CRA) is considered to be associated with multiple pathological and clinical factors, such as bleeding, nutritional deficiency, and bone marrow suppression [6]. Bone marrow suppression can be caused by both malignant cell infiltration and chemotherapy treatment [7, 8]. Functional iron deficiency is usually associated with insufficient iron intake because of cancer-related appetite loss and bleeding [9, 10]. At present, the treatments of anaemia and cancer are complementary. Under these circumstances, it is critical to identify the association of relevant elements, including clinicopathological characteristics and GC treatment model, with anaemia in recurrent or metastatic GC.

Our study aimed to determine the role of initial degree of anaemia and cancer-related haemoglobin reduction in the prognosis of recurrent or metastatic GC patients. The relationships between clinicopathological characteristics, including treatment regimens, and cancer-related haemoglobin reduction degree were further analysed. Our study will contribute to the determination of treatment approaches for recurrent or metastatic GC-related anaemia patients.

## Methods

### Patients

All procedures followed were in accordance with the ethical standards of the ethical committee of Shandong Provincial Hospital regarding human experimentation and with the 1964 Helsinki Declaration and later versions. Informed consent for inclusion in the study was obtained from all patients.

Our retrospective study analysed the data collected from patients diagnosed with metastatic GC or recurrent GC at Shandong Provincial Hospital in China from January 1, 2010, to December 31, 2014. The entry criteria included the following: 1) metastatic GC or recurrent GC after radical surgical treatment was histologically confirmed as gastric adenocarcinoma — radical gastric resection was defined as negative margins, en bloc resection of the greater and lesser omentum, and D2 lymph node dissection, and standard lymphadenectomy was defined as when the number of retrieved lymph nodes was ≥15; 2) The Eastern Cooperative Oncology Group performance score (ECOG PS) was used to estimate a life expectancy of more than 3 months [11]; and 3) patients had received at least one cycle of chemotherapy. The exclusion criteria included the following: 1) accompaniment by other types of malignancies, 2) use of neoadjuvant chemotherapy, and 3) loss to follow-up. All the pathologic specimens were reviewed by at least 2 pathologists to confirm the diagnosis of GC.

### Haemoglobin level measurement

The initial haemoglobin level (HB_ini_) was collected at the initial diagnosis of recurrent or metastatic GC. The lowest haemoglobin level was determined as the lowest level obtained from the day of diagnosis to the date of death or the final follow-up visit. The decrease in haemoglobin (HB_dec_) was defined by subtracting the lowest haemoglobin level from the initial haemoglobin level. Evaluation and grading of anaemia were performed according to National Comprehensive Cancer Network (NCCN) guidelines for cancer- and chemotherapy-induced anaemia [12].

When the HBini was less than 70 g/L, RBC transfusions were used to improve the anaemia until the initial Hb was more than 70 g/L, and the dose of chemotherapeutic drugs was not regulated.

### Chemotherapy regimens

The regimens used to treat the patients included the combination chemotherapy of docetaxel, cisplatin, and 5-fluorouracil (DCF) and related modifications (docetaxel 75 mg/m^2^ on day 1, cisplatin 60 mg/m^2^ or oxaliplatin 130 mg/m^2^ on day 1, fluorouracil 2500 mg/m^2^ continuous infusion 120 h, cycled every 21 days); XP or modifications (capecitabine 1000 mg/m^2^ twice daily (BID) on days 1–14, cisplatin 75 mg/m^2^ or oxaliplatin 130 mg/m^2^ on day 1); FOLFIRI (irinotecan 180 mg/m^2^ on day 1, leucovorin 400 mg/m^2^ on day 1, fluorouracil 400 mg/m^2^ IV push on day 1, fluorouracil 2400 mg/m^2^ continuous infusion 46 h, cycled every 14 days); paclitaxel liposome 100 mg/m^2^ (q2w) or 135–150 mg/m^2^ (q3w) on day 1, combine with capecitabine or S-1; and single agents such as docetaxel 75–100 mg/m^2^ on day 1, capecitabine 1000–1250 mg/m^2^ BID on days 1–14, or S-1 80–120 mg on days 1–14, cycled every 21 days. The first line chemotherapy regimens include DCF, Paclitaxel liposome + Capecitabine / S-1 or XP. The second line chemotherapy regimens include FOLFIRI or single agent. The treatment effect of chemotherapy was estimated after 2 cycles of the chemotherapy regimen with 3 weeks or 3 cycles of the chemotherapy regimen with 2 weeks.

### Follow-up

Tumour responses to the chemotherapy regimens were evaluated after every 2–3 cycles of chemotherapy and categorized based on the Response Evaluation Criteria in Solid Tumors (RECIST) 1.1 guidelines [13]. The number of malignant ascites and peritoneal cytology were also considered when assessing the antitumour effects.

Overall survival (OS) was calculated as the time from the date of initial diagnosis of metastatic GC or the date of recurrence after GC resection to the date of either death or the final follow-up. Progression-free survival (PFS) was calculated as the date of either disease progression, confirmed by magnetic resonance imaging or computed tomography using a contrast medium if possible, or death from any cause.

Clinical variables for risk assessment consisted of patient demographics, surgical and pathological factors, chemotherapy regimens, and packed red cell transfusion. Data regarding recurrence, defined as disease recurrence at any site, and survival outcomes were also collected.

Peritoneal metastasis is a frequent type of metastasis of gastric cancer and is a definitive determinant for prognosis. Peritoneal metastasis was diagnosed by histological diagnosis of peritoneal metastasis and/or by peritoneal lavage cytology positive for cancer cells.

### Statistical analysis

Survival analyses were performed by Kaplan-Meier curves with log-rank tests for significance. Statistical analysis included univariate analysis and multivariate analysis. Univariable Cox regression analyses were performed using PFS, OS and HB_dec_ as the outcomes, with a significance level of *p* < 0.05. Multivariate analysis was carried out with a Cox proportional hazards model to evaluate prognostic factors with respect to PFS, OS and HB_dec_. The factors which were potential risk factors for GC patient’s prognosis or having statistical significance from the univariate analysis data were performed in Cox multivariate model. Hazard ratios (HRs) and 95% confidence intervals (CIs) were calculated. A value of *p* < 0.05 was considered statistically significant. All statistical analyses were conducted using SPSS statistical software (Version 24.0; IBM Corporation, Armonk, NY, USA).

## Results

### Patients

Based on the inclusion and exclusion criteria, 598 patients were included in our study. Our study included 170 recurrent GC patients and 428 metastatic GC patients. The general characteristics including the kinds of chemotherapy regimen of all enrolled patients are listed in Table 1. The age and gender proportions and surgical and pathological factors of the patient population were similar to those observed in other studies [14].

**Table 1:** Patients characteristics

	Total *N* = 598	
**Age**		
< 65 years, N (%)	230(38.5)	
≥ 65 years, N (%)	368(61.5)	
**Gender**		
Male, N (%)	469(78.4)	
Female, N (%)	129(21.6)	
**Palliative setting**		
Initially metastatic	428(71.6)	
Recurrent	170(28.4)	
**Operation method**		
Proximal gastrectomy	60(35.3)	
Distal gastrectomy	83(48.8)	
Total gastrectomy	27(15.9)	
**Pathological type**		
Well differentiated	4(0.7)	
Moderately differentiated	59(9.8)	
Poorly differentiated	250(41.8)	
Signet ring cell	61(10.2)	
Unassorted	224(37.5)	
**Fecal occult blood#**		
Positive	129(33.9)	
Negative	381(74.7)	
**Combination of three regimens**	217(36.3)	
**Treatment response**	*N* = 312	
Partial response	14(4.49)	
Stable disease	169(54.17)	
Progressive disease	129(41.35)	
**Tumor location**		
Upper part (U)	253(42.3)	
Middle part (M)	91(15.2)	
Lower part (L)	206(34.4)	
ML	29(4.8)	
MU	19(3.2)	
**T/N stage**		
Ia + Ib	4 + 7(6.5)	
IIa + IIb	8 + 15(13.5)	
IIIa+IIIb+IIIc	34 + 46 + 56(80.0)	
**Hemoglobin level (g/L)**	**Initial**	**Post-treatment**
> 110	398	327
100–110	78	91
80–100	82	124
65–80	26	41
< 65	14	15
**Manifestations of tumor hemorrhage**	**Initial**	**During treatment**
Fecal occult blood +	37	47
Erosion and bleeding by endoscopy	92	76
Hematemesis	21	40
Iron deficiency anemia	3	9
chemotherapy-induced anemia	0	136
Unknown	50	26
DIC	0	5
Bone marrow infiltration	0	4
**Chemo regimens**		
DCF	217	
FOLFIRI	152	
Paclitaxel liposome+Capecitabine/S-1	207	
XP	264	
Single agent	215	

# Fecal occult blood: 88 patients not testing at the date of diagnosis

There were 312 patients treated with the first line chemotherapy regimens, yet the GC in 188 patients remained in a development condition, and then those patients were treated with the second or/and third line chemotherapy regimens including FOLFIRI and docetaxel single agent. The cycles of chemotherapy used for our GC patients was 4.4 ± 3.705 [1–20]. Two hundred eighty six patients (47.8%) failed to receive further chemotherapy after 1–2 cycles of chemotherapy treatment.

### Follow-up and survival

Of the 598 GC patients, the median follow-up time was 11.60 months (range 0–76), and the median OS after chemotherapy was 12 months (95% CI 11.221–12.779), with 1-, 3-, and 5-year OS rates of 45.40, 3.80, and 0.90%, respectively.

The 598 patients were divided into the HB_ini_ ≤ 80 g/L cohort and the HB_ini_ level > 80 g/L cohort. Our study included 40 patients in the HB_ini_ ≤ 80 g/L cohort and 558 patients in the HB_ini_ level > 80 g/L cohort. The clinical features which have potential effects on GC patient OS and PFS were well matched between our two groups (Table 2).

**Table 2:** Clinical features which have potential effects on GC patient’s OS and PFS

	HB_ini_ ≤ 80 g/L	HB_ini_ > 80 g/L	*p* value
Liver metastasis	17	229	0.856
Bone metastasis	1	24	0.888
Peritoneal metastasis	1	66	0.122
Lung metastasis	5	94	0.475
Metastatic sites ≥3	11	116	0.316

**Fig. 1:**
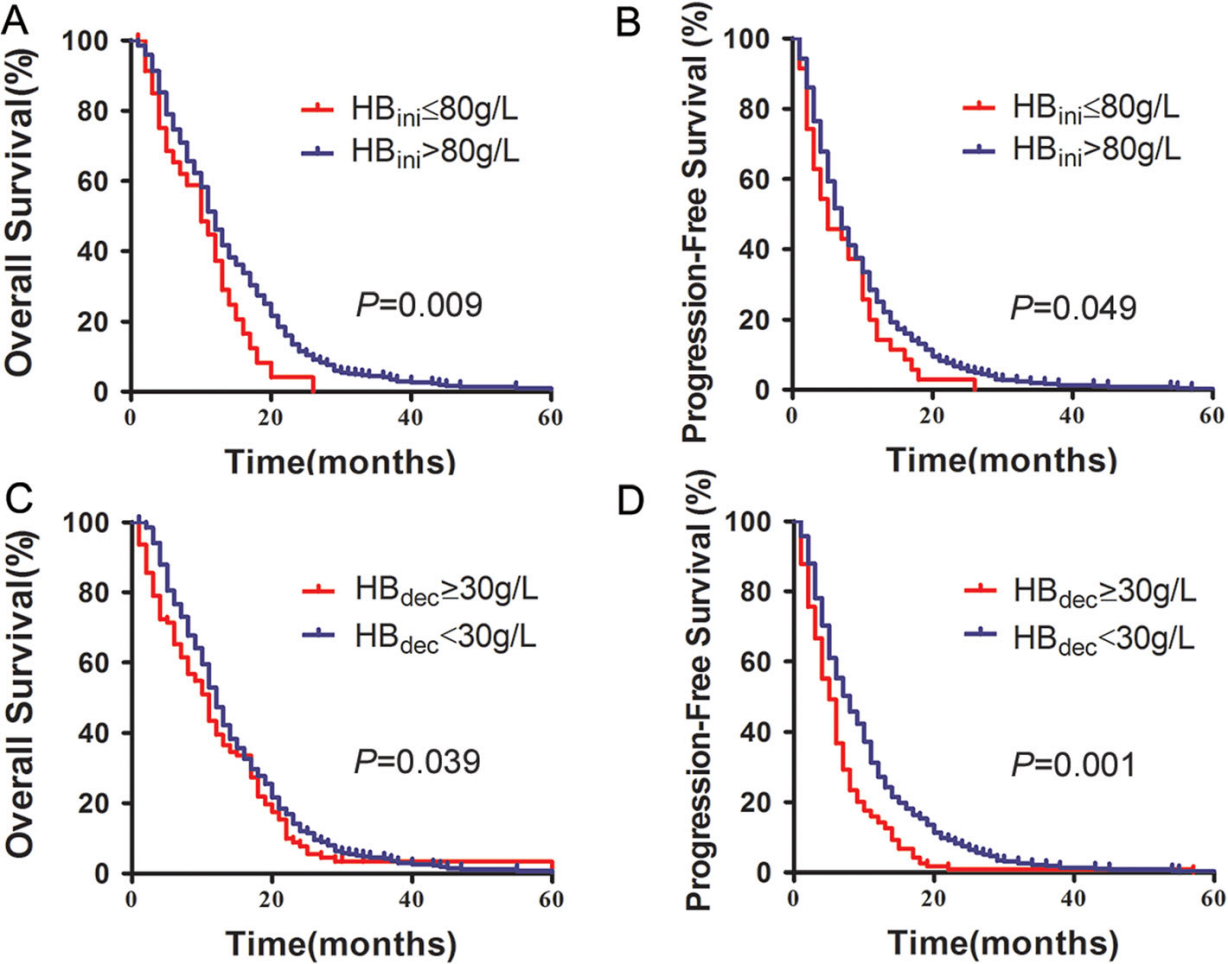
The OS and PFS curves for 598 patients according to the degree of anaemia. **a**. OS curve according to HB_ini_ level (40 patients in the HB_ini_ ≤ 80 g/L cohort and 558 patients in the HB_ini_ level > 80 g/L cohort); **b**. PFS curve according to HB_ini_ level (40 patients in the HB_ini_ ≤ 80 g/L cohort and 558 patients in the HB_ini_ level > 80 g/L cohort); **c**. OS curve according to HB_dec_ (53 patients in the HB_dec_ ≥ 30 g/L cohort and 545 patients in the HB_dec_ < 30 g/L cohort); **d**. PFS curve according to HB_dec_ (≥ 30 g/L and < 30 g/L)

For the HB_ini_ ≤ 80 g/L cohort, the median OS was 10 months with 1-, 3-, and 5-year survival rates of 35.40, 0, and 0%, respectively, while in the HB_ini_ level > 80 g/L cohort, the median OS was 12 months with 1-, 3-, and 5-year survival rates of 46.10, 4.10, and 3.00%, respectively. The OS of the HB_ini_ ≤ 80 g/L cohort was significantly worse than that of the HB_ini_ level > 80 g/L cohort (*p* = 0.009, Fig. 1a, Table 3).

**Table 3:** Median OS and PFS

Variable	Median OS (m)	95% CI	*p* value	Median PFS (m)	95% CI	*p* value
HB_ini_ ≤ 80 g/L	10.0	6.147–13.853	*0.009*	5.0	3.038–6.962	0.049
HB_ini_ > 80 g/L	12.0	11.191–12.809		7.0	6.532–7.648	
HB_dec_ ≥ 30 g/L	11.0	8.899–13.101	0.039	5.0	4.097–5.903	0.001
HB_dec_ < 30 g/L	12.0	11.151–12.849		7.0	6.059–7.941	
Transfusion yes	12.0	9.436–14.564	0.769	6.0	4.495–7.505	0.468
Transfusion no	12.0	11.185–12.815		7.0	6.266–7.734	

Then, we compared the OS and PFS between the HB_ini_ ≤ 80 g/L cohort and the cohort with HB_ini_ between 80 g/L and 110 g/L. Our results revealed that the HB_ini_ ≤ 80 g/L cohort did not have a trend of worse OS and PFS than the mild anaemia cohort (Supplementary Table 1).

Kaplan-Meier analysis was also used to analyse the correlation between HB_ini_ level and PFS. Our results revealed that patients with HB_ini_ levels ≤80 g/L also had a trend toward a shortened median PFS (*p* = 0.049, Fig. 1b, Table 3). Interestingly, we also found that HB_dec_ ≥ 30 g/L was associated with a significantly shortened median OS (*p* = 0.039, Fig. 1c), and a similar relationship was found with decreased median PFS (*p* = 0.001, Fig. 1d, Table 3).

Red blood cell (RBC) transfusion is an important treatment modality, while chemotherapy is beneficial for improving the prognosis of recurrent and metastatic GC patients. We analysed the different treatment modalities and clinicopathological parameters for the OS and PFS in our patients.

Using univariate analysis, we found that RBC transfusion was associated with neither median OS nor median PFS. The factors that significantly influenced OS were HB_ini_ level, HB_ini_ ≤ 80 g/L, metastatic sites ≥3, liver metastases, paclitaxel-based combination of three regimens, the number of chemotherapy cycles, treatment response, and HB_dec_ ≥ 30 g/L (*p* < 0.05). Additionally, HB_ini_ level, the lowest haemoglobin level, metastatic sites ≥3, liver metastases, bone metastases, number of chemotherapy cycles, chemotherapy including paclitaxel, treatment response and HB_dec_ ≥ 30 g/L were significantly associated with PFS (*p* < 0.05) (Table 4).

**Table 4:** Univariate analyses of risk factors for OS and PFS

	N = 598	OS				PFS		
		*p* value	HR	95%CI		*p* value	HR	95%CI
HB_ini_		0.010	0.995	0.991–0.999		0.013	0.995	0.992–0.999
HB_lowest_		0.575	0.999	0.995–1.003		0.010	0.995	0.992–0.999
HB_ini_ ≤ 80 g/L	40(6.7)	0.012	1.608	1.109–2.332		0.065	1.371	0.981–1.916
HB_ini_ > 80 g/L	558(93.3)							
Metastases								
Metastatic sites ≥3	127(21.2)	0.033	1.268	1.020–1.577		0.015	1.289	1.050–1.583
Metastatic sites < 3	471(78.8)							
Lymph node	457(76.4)	0.849	0.980	0.794–1.209		0.276	0.896	0.734–1.092
Liver	246(41.1)	0.010	1.271	1.059–1.525		0.001	1.354	1.141–1.607
Lung	99(16.6)	0.399	0.899	0.703–1.151		0.221	1.150	0.920–1.438
Bone	25(4.2)	0.072	1.495	0.964–2.318		0.017	1.651	1.093–2.493
Peritoneum	67(11.20)	0.181	0.821	0.651–1.096		0.771	0.960	0.729–1.264
Chemotherapy regimen								
Included paclitaxel	239(40.0)	0.116	1.160	0.964–1.397		0.018	1.232	1.037–1.463
Included platinum	61(10.2)	0.290	0.849	0.626–1.150		0.734	0.985	0.748–1.296
Number of cycles		< 0.001	0.916	0.894–0.940		0.006	0.97	0.948–0.991
Number of PTX3*		0.023	0.937	0.885–0.991		0.940	1.002	0.955–1.051
Treatment response								
Progressive disease	188(31.4)	0.041	1.223	1.008–1.484		< 0.001	1.959	1.634–2.350
Non-progressive disease	410(68.6)							
HB_dec_								
≥ 30	131(21.9)	0.048	1.244	1.002–1.546		< 0.001	1.594	1.302–1.951
< 30	467(78.1)							
Transfusion	87(14.5)	0.778	1.038	0.802–1.342		0.492	1.085	0.860–1.367
No transfusion	511(85.5)							
Adjuvant chemotherapy	170	0.735	1.010	0.954–1.070		0.470	0.981	0.931–1.034

*PTX3 paclitaxel-based combination of three regimens

Multivariate analysis showed that HB_ini_ level ≤ 80 g/L (HR = 1.879, 95% CI = 1.301–2.767, *p* = 0.001), liver metastases (HR = 1.234, 95% CI = 1.022–1.490, *p* = 0.029), chemotherapy including paclitaxel (HR = 1.225, 95% CI = 1.013–1.481, *p* = 0.036), treatment response (HR = 1.457, 95% CI = 1.173–1.808, *p* = 0.001), and HB_dec_ ≥ 30 g/L (HR = 1.536, 95% CI = 1.206–1.957, *p* = 0.001) were significant adverse prognosis factors of OS. More importantly, the number of chemotherapy cycles was also significantly correlated with improved OS (HR = 0.879, 95% CI = 0.855–0.904, *p* < 0.001) (Table 5).

**Table 5:** Multivariate analyses of risk factors for OS and PFS

	OS			PFS		
	*p* value	HR	95%CI	*p* value	HR	95%CI
HB_ini_ ≤ 80 g/L	0.001	1.879	1.301–2.767	0.016	1.516	1.082–2.126
metastatic sites ≥3	0.063	1.246	0.989–1.572	0.823	1.026	0.821–1.281
Liver metastases	0.029	1.234	1.022–1.490	0.057	1.188	0.885–1.420
Bone metastases	0.269	1.293	0.820–2.040	0.685	1.094	0.709–1.689
Chemotherapy included paclitaxel	0.036	1.225	1.013–1.481	0.007	1.273	1.068–1.517
Number of cycles	< 0.001	0.879	0.855–0.904	< 0.001	0.922	0.899–0.945
Treatment response	0.001	1.457	1.173–1.808	< 0.001	2.235	1.818–2.747
HB_dec_ ≥ 30 g/L	0.001	1.536	1.206–1.957	< 0.001	1.543	1.233–1.932

For PFS, HB_ini_ level ≤ 80 g/L (HR = 1.516, 95% CI = 1.082–2.126, *p* = 0.016), chemotherapy including paclitaxel (HR = 1.273, 95% CI = 1.068–1.517, *p* = 0.007), treatment response (HR = 2.235, 95% CI = 1.818–2.747, p < 0.001), the number of chemotherapy cycles (HR = 0.922, 95% CI = 0.899–0.945, *p* < 0.001), and HB_dec_ ≥ 30 g/L (HR = 1.543, 95% CI = 1.233–1.932, *p* < 0.001) were independent prognostic factors (Table 5).

**Fig. 2:**
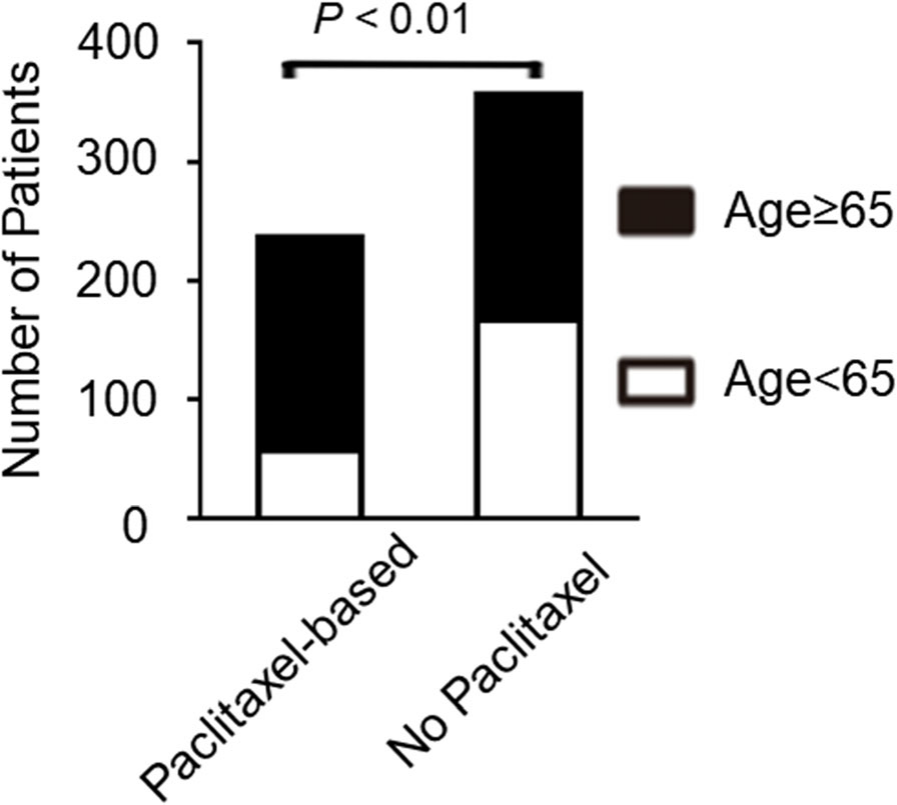
Patients who received paclitaxel-based chemotherapy were older than those who did not receive paclitaxel

To further determined the reason why chemotherapy including paclitaxel could influence the prognosis in our cohort patients, we analyzed the difference of clinical characteristics between patients who received chemotherapy including paclitaxel and those who did not. Our results revealed that the patients who received chemotherapy including paclitaxel were older than those who did not receive paclitaxel including chemotherapy in our cohort (Fig. 2) .

### Relationship between the degree of decrease in haemoglobin levels and the clinicopathological parameters of our patients

We then investigated whether we could identify correlations between the degree of decrease in haemoglobin levels and the clinicopathological parameters of our GC patients. Our results suggested that bone metastases, chemotherapy including platinum, the number of chemotherapy cycles, and treatment response were associated with the degree of haemoglobin decrease (*p* < 0.05) (Table 6). Multivariate analyses revealed that the degree of HB_dec_ were significantly correlated with the number of chemotherapy cycles and chemotherapy including platinum (*p* < 0.001 and *p* = 0.019, respectively), and was not relevant with chemotherapy included paclitaxel (Table 7).

**Table 6:** Univariate analyses of risk factors for HB_dec_

	*p* value	HR	95%CI
Metastases			
metastatic sites ≥3	0.141	0.838	0.663–1.060
metastatic sites < 3			
Metastatic site			
Lymph node	0.325	1.131	0.885–1.444
Liver	0.328	1.107	0.903–1.358
Lung	0.936	0.989	0.763–1.283
Bone	0.017	0.574	0.365–0.905
Chemotherapy regimen			
Included paclitaxel	0.876	0.984	0.802–1.207
Included platinum	0.010	0.645	0.463–0.899
Number of cycles	< 0.001	0.933	0.907–0.961
Number of PTX3*	0.161	0.96	0.908–1.016
Treatment response			
Progressive disease	0.037	0.798	0.646–0.986
Non-progressive disease

**Table 7:** Multivariate analyses of risk factors for HB_dec_

Variable	*p* value	HR	95% CI
Chemotherapy included platinum	0.019	0.661	0.468–0.934
Metastatic sites ≥3	0.371	0.895	0.702–1.141
Bone metastases	0.055	0.633	0.396–1.010
Chemotherapy included paclitaxel	0.061	1.226	0.991–1.517
Number of chemotherapy cycles	< 0.001	0.938	0.911–0.966
Liver metastases	0.060	1.227	0.991–1.520
Treatment response	0.111	0.833	0.665–1.043

Chemotherapy drugs can not only kill cancer cells, but also damage healthy cells, which causes side effects. Our results revealed that the most common side effects of chemotherapy were myelosuppression, diarrhea and vomiting, yet which could not influence the OS and PFS in our cohort (Table 8).

**Table 8:** Univariate analyses of chemotherapy side effects for OS and PFS

	N = 598	OS				PFS		
		*p* value	HR	95%CI		*p* value	HR	95%CI
Myelosupression								
Degree I	103	0.001	3.117	1.809–4.371		0.148	1.47	0.872–2.48
Degree II	137	0.075	1.371	0.911–2.943		0.598	1.157	0.372–1.993
Degree III	63	0.102	1.591	0.911–2.776		0.680	1.119	0.656–1.909
Degree IV	15	0.998	1.001	0.552–1.814		0.600	0.860	0.489–1.512
Diarrhea								
Grade1	519							
Grade2	52	0.196	0.757	0.496–1.155		0.227	0.787	0.533–1.161
Grade3	27	0.045	0.580	0.40–0.989		0.313	0.785	0.490–1.256
Vomiting								
Grade1	136							
Grade 2	443	0.189	0.702	0.414–1.190		0.738	0.917	0.553–1.521
Grade 3	19	0.213	0.727	0.440–1.201		0.252	0.752	0.462–1.225

## Discussion

CRA occurs as a result of multiple aetiologies, including blood loss, functional iron deficiency, erythropoietin deficiency due to renal disease, chemotherapy-induced myelosuppression, marrow involvement with tumours and other factors. The relationship between anaemia and the prognosis of GC patients is rarely reported. Zhang et al. reported that patients with less than ≤65 g/L haemoglobin had a significantly shorter median OS than patients with 65 g/L to normal haemoglobin or patients with normal haemoglobin and demonstrated that a lower haemoglobin level might predict poorer OS in advanced GC patients [15]. There is little information to evaluate the effect of anaemia status and RBC transfusion treatment on the OS and PFS of recurrent or metastatic GC patients.

According to the NCCN guidelines for cancer- and chemotherapy-induced anaemia, a haemoglobin level ≤ 80 g/L is used to define severe-grade anaemia. Our present study also chose a haemoglobin level of 80 g/L as the cut-off value for severe anaemia. Our results revealed that pretreatment of severe anaemia could serve as a prognostic factor in metastatic GC or recurrent GC patients who underwent radical resection and were then treated with chemotherapy. Multivariate analysis also showed that an initial haemoglobin level ≤ 80 g/L was an independent adverse prognostic factor for our patients. In addition, the degree of haemoglobin decrease (haemoglobin level ≥ 30 g/L) during chemotherapy or the follow-up period was also an important risk factor for the prognosis of recurrent or metastatic GC.

The cause of anaemia in patients with cancer is often multifactorial. The malignancy itself can lead to or exacerbate anaemia, and underlying comorbidities may also contribute to anaemia. Cancer cells can directly suppress haematopoiesis through bone marrow infiltration and produce cytokines, leading to iron sequestration. Chronic blood loss, nutritional deficiencies, myelosuppressive effects of chemotherapy, and radiation therapy to the skeleton can further exacerbate anaemia in patients with cancer [6–10]. Due to the potentially multifactorial complexity of anaemia, defining the causes of anaemia in cancer patients is essential, which will contribute to determining the appropriate treatment method to apply.

Previous researches recognized paclitaxel as first- or second-line chemotherapy, in which median overall survival was several months [16, 17]. Our study revealed that paclitaxel was an independent adverse prognostic factor, but was not relevant with the degree of HB_dec_. Our results were also different with previous research which showed that docetaxel, a newly taxoid anticancer drug, can cause a progression in anaemia from grade III to IV in 9% of patients [18]. The reasons for those results may be related with the proportion of elderly patients. Thus, the role of paclitaxel in influencing the prognosis and HB level of the GC patient needs for further assessment.

To the contrary, our results showed that chemotherapy including platinum was associated with a decrease in haemoglobin in recurrent or metastatic GC patients, which are similar to the findings of previous reports. Groopman et al. reported that platinum-based regimens are well known to induce anaemia due to the combined bone marrow and kidney toxicity, and the use of chemotherapy regimens including paclitaxel is an adverse prognostic factor for decreased haemoglobin, although this effect is not significant [19]. Therefore, we consider that platinum should not be recommended to treat severely anaemic recurrent or metastatic GC patients until the anaemia has been improved through treatment. The other regimens such as capecitabine can be chose to treat the severely anaemic GC patients.

The most common treatment options for CRA include erythropoietic-stimulating agents, RBC transfusion and nutritional therapy, such as iron intake. Previous studies have reported that the lowest postoperative haemoglobin level and postoperative transfusion were the most significant risk factors for postoperative complications in GC surgery [20]. Squires et al. reported that perioperative allogeneic blood transfusion was associated with decreased PFS and OS after resection of GC, independent of adverse clinicopathologic factors [21]. In addition, RBC transfusion could not improve the chemotherapy outcomes by increasing the haemoglobin level [22]. However, the role of RBC transfusion in improving the prognosis of recurrent or metastatic GC patients remains unclear. Our present data support the notion that transfusion neither significantly improved the OS and PFS nor served as a risk factor for PFS and OS in recurrent or metastatic GC. These results may be attributed to the fact that transfusion was used only when haemoglobin was not more than 70 g/L in our hospital. Insufficient blood transfusion may be another possible reason for this result.

However, our study also has several limitations. First, our study is a retrospective analysis, so our results should be confirmed by multicenter-randomized trials. Second, the inequality in the number of patients enrolled in our different cohorts can also generate potential bias. Third, high proportion of patients (286/598, 47.8%) only received 1–2 cycles of chemotherapy treatment, which can also influence the treatment effect of chemotherapy.

## Conclusions

Our study demonstrated that the initial degree of anaemia can serve as a biomarker for predicting the prognosis of recurrent or metastatic GC patients, while chemotherapy treatment rather than RBC transfusion can improve OS and PFS. In addition, platinum should not be recommended to treat severely anaemic GC patients.

## Supplementary information

**Additional file 1 Table S1.** Median OS and PFS

## Data Availability

The datasets used and/or analyzed during the current study available from the corresponding author on reasonable request.

## Abbreviations

GC: Gastric cancer
CRA: Cancer-related anaemia
ECOG PS: The Eastern Cooperative Oncology Group performance score
HB_ini_: The initial haemoglobin level
HB_dec_: The decrease of haemoglobin
NCCN: National Comprehensive Cancer Network
DCF: Docetaxel, cisplatin, and 5-fluorouracil
BID: Twice daily
RECIST: Response Evaluation Criteria in Solid Tumors
OS: Overall survival
PFS: Progression-free survival
HRs: Hazard ratios; RBC, Red blood cell
