# Supplementary material for: Role of the initial degree of anaemia and treatment model in the prognosis of gastric cancer patients treated by chemotherapy: a retrospective analysis
Source: BMC Cancer. 2020 May 13;20:414. doi: 10.1186/s12885-020-06881-7 (PMC7222574; doi:10.1186/s12885-020-06881-7)
Supplement: Supplementary file 1 — Additional file 1 Table S1. Median OS and PFS [file 12885_2020_6881_MOESM1_ESM.docx]

Table S1 Median OS and PFS

| Variable | Median OS (m) | 95% CI | *p* value | Median PFS (m) | 95% CI | *p* value |
| --- | --- | --- | --- | --- | --- | --- |
| HB_ini_ ≤80 g/L | 10.0 | 6.147-13.853 |  | 5.0 | 3.038-6.962 |  |
| HB_ini_ 80-110 g/L  HB_ini_ >110 g/L | 11.0  12.0 | 9.402-12.598  11.010-12.779 | 0.128  *0.003* | 5.0  7.0 | 4.117-5.883  6.170-7.830 | 0.419  *0.017* |
